# Supplementary material for: Fetal Growth Associated with Maternal Rheumatoid Arthritis and Juvenile Idiopathic Arthritis
Source: Healthcare (Basel). 2024 Nov 28;12(23):2390. doi: 10.3390/healthcare12232390 (PMC11641325; doi:10.3390/healthcare12232390)
Supplement: Supplementary file 1 [file healthcare-12-02390-s001.zip › healthcare-3300365-supplementary.pdf]

Fetal Growth Associated with Maternal Rheumatoid Arthritis and Juvenile Idiopathic Arthritis.

Supplementary Material

Table S1. ICD-10 codes to identify women who were diagnosed with RA and JIA prior to, and during their pregnancies.

Condition	ICD-10 code(s)
Rheumatoid Arthritis	M05.X–M06.X
Juvenile Idiopathic Arthritis	M08.X

Table S2. Anatomical Therapeutic Codes of Antirheumatic Therapies (ART) that were retrieved from the Danish National Prescription Registry (NPR). *

Medications	ATC Code(s)
Glucocorticoids	H02AB
Prednisone	H02AB07
Betamethasone	H02AB01
Hydrocortisone	H02AB09
Non-steroidal anti-inflammatory drugs	M01A
Methotrexate	L01BA01, L04AX03
Hydroxychloroquine	P01BA02
Leflunomide	L04AA13
Sulfasalazine	A07EC01

Table S3. Estimated fetal weights and birth weights of offspring among women with and without RA/JIA.

Estimated Fetal Weight (Z-score) During Second Trimester								Birth Weight (Z-score)							
	Mean value	Crude model			Adjusted model**				Mean value	Crude model			Adjusted model**		
		Mean Difference *	95% CI	p-value	Mean Difference	95% CI	p-value			Mean Difference *	95% CI	p-value	Mean Difference	95% CI	p-value
No RA/JIA (Ref)	Ref	-	-	-	-	-	-	No RA/JIA	Ref	-	-	-	-	-	-
RA/ JIA	0.07 (0.02, 0.11)	0.05	0.00, 0.09	**0.031**	0.05	0.01, 0.10	**0.022**	RA/ JIA	-0.14 (-0.19, -0.10)	-0.07	-0.11, -0.03	**0.001**	-0.08	-0.13, -0.04	**<0.001**
**Antirheumatic medications use during pregnancy among women with RA/JIA compared with the reference group***															
CCS±	-0.03 (-0.16, 0.10)	-0.05	-0.18, 0.08	0.5	-0.05	-0.18, 0.08	0.5	CS	-0.35 (-0.49, -0.21)	-0.28	-0.40, -0.15	**<0.001**	-0.31	-0.43, -0.18	**<0.001**
HCQ	-0.03 (-0.27, 0.20)	-0.05	-0.27, 0.36,	0.8	-0.02	-0.34, 0.31	>0.9	HCQ use	-0.29 (-0.59, 0.01)	-0.22	-0.53, 0.09	0.2	-0.27	-0.59, 0.04	0.091
SSZ	0.39 (0.25, 0.53)	0.37	0.24, 0.50	**<0.001**	0.38	0.24, 0.51	**<0.001**	SSZ	-0.26 (-0.39, -0.12)	-0.18	-0.32, -0.05	**0.006**	-0.23	-0.37, -0.10	**<0.001**

HCQ: hydroxychloroquine; SSZ: sulfasalazine

± Corticosteroids (CCS): betamethasone, prednisone, and hydrocortisone.

Reference group*: women without RA/JIA

* Mean difference in Z-scores between women with RA/JIA and women without RA/JIA (reference group).

** Model adjusted for maternal age, maternal BMI, smoking status, income status, race, parity, birth year, pre-pregnancy hypertension, pre-pregnancy diabetes, and co-medication use.

Table S4. Effect analysis of the influence of rheumatoid arthritis (RA)/ juvenile idiopathic arthritis (JIA) and pre-eclampsia, as a mediator on small for gestational age (SGA).

Mediator	Total Effect Odds Ratio (OR)*	Controlled Direct Effect OR**	Proportion Mediated (%) ¶
Pre-eclampsia ±	1.47 (1.13-1.79)	1.45 (1.22 – 1.83)	4.23

* Effect of RA/JIA on SGA

** Effect of RA/JIA on SGA that is not mediated by pre-eclampsia

¶ Effect of RA/JIA on SGA mediated by pre-eclampsia

Proportion of the effect of RA/JIA on SGA, mediated by pre-eclampsia: calculated as log(indirect effect)/log(total effect)*100

± Adjusted for age, BMI, smoking status, income status, race, and parity (+ pre-gestational hypertension and pre-gestational diabetes between mediator and outcome)
